# Editorial: The role of TNF-TNFR2 signal in immunosuppressive cells and its therapeutic implications, volume II

**DOI:** 10.3389/fimmu.2023.1227003

**Published:** 2023-08-01

**Authors:** Xin Chen, Magdalena Plebanski

**Affiliations:** ^1^ State Key Laboratory of Quality Research in Chinese Medicine, Institute of Chinese Medical Sciences, University of Macau, Macau, Macau SAR, China; ^2^ School of Health and Biomedical Sciences, RMIT University, Bundoora, VIC, Australia

**Keywords:** TNF, TNFR2, CD4^+^Foxp3^+^ regulatory T cells, inflammation, cancer, autoimmune diseases

## Introduction

There is compelling evidence that TNFR2 signaling plays a decisive role in the activation, expansion, function and phenotypical stability of CD4^+^Foxp3^+^ regulatory T cells (Tregs) (1–4). Current research focuses on the translation of this property of TNFR2 into novel treatment for major human diseases, while the molecular basis underlying Treg-stimulatory effect of TNFR2 is also actively pursued. This Research Topic showcases the latest experimental studies as well as expert perspectives incorporating recent findings in the literature.

## Inhibition of TNFR2 signaling for cancer immunotherapy

Chen, Y. et al. from Adlai Nortye USA Inc. reported that a humanized antagonistic antibody, AN3025, at sub-nanomolar affinity binds to the extracellular domain of human TNFR2. *In vitro* treatment with AN3025 inhibited the immunosuppressive effect of human Tregs (hTregs), resulting in an enhanced proliferation and IFNγ production by co-cultured effector T cells (Teffs). In TNFR2 humanized mice, AN3025 treatment markedly inhibited the growth of MC38 tumor, accompanied by the reduction of tumor-infiltrating Tregs and an increase in the number of effector CD4 and CD8 T cells in the tumor. Further, the *in vivo* anti-tumor effect of AN3025 could be enhanced when used together with anti-PD-L1 antibody. Thus, this antagonistic anti-TNFR2 antibody may be used as a monotherapy or combined with other immune therapeutics in the treatment of cancer (Chen, Y. et al.)

## Levels of soluble TNFR2 in the circulation as a cancer diagnostic biomarker

Based on the previously published data, Kartikasari et al. from RMIT University analyzed the correlation of levels of soluble TNFR2 (sTNFR2) in the blood and the risk of cancer. The results show that the levels of sTNFR2 were markedly enhanced in circulation of patients with spectrum of cancers, including colorectal cancer, ovarian cancer, breast cancer, non-Hodgkin’s lymphoma, Hodgkin’s lymphoma, lung cancer, hepatocarcinoma, and glioblastoma. A random-effect meta-analysis shows that the cancer-specific odds ratio (OR) significantly correlated between the elevated levels of circulating sTNFR2 and the risk of colorectal cancer, non-Hodgkin’s lymphoma, and hepatocarcinoma. Consistent with the notion that TNFR2 promotes the development of cancer, this study for the first time provides clear evidence that sTNFR2 in the circulation may be used as a diagnostic biomarker for certain types of cancers (Kartikasari et al.).

## Activation of TNFR2 signaling for the treatment of inflammatory diseases

Previously, it was shown that the clinical effect of anti-TNF antibody (adalimumab) on rheumatoid arthritis is at least partially mediated by the elevated levels of transmembrane bound TNF expression on monocytes and the expansion of Tregs through TNFR2 signaling (5). This may represent a common mechanism of some anti-inflammatory or immunosuppressive therapeutics. Since tetrandrine (TET), a small molecular compound isolated from Chinese herb medicine, was found to have similar effect as adalimumab (6). Chen, S. et al. from University of Macau explored the possibility of transforming their study on TET into a treatment for psoriasis. To this end, TET nanoemulsion was topically administered on a mouse model of psoriasis, and this treatment resulted in the expansion of TNFR2-expressing Tregs and inhibition of skin inflammation in WT or TNFR1 KO mice, but not in TNFR2 KO mice (Chen, S. et al.).

Lambuk et al. from Universiti Sains Malaysia systemically analyzed the literature regarding the role of TNF, TNFR1 and TNFR2 in glaucomatous neurodegeneration. They concluded that selectively targeting certain TNF receptors may represent a more effective and safer strategy in the treatment of glaucoma, as compared with the global blockage of TNF, given TNFR2 activation of Tregs can exhibit a potent immunosuppressive effect (Lambuk et al.).

Vargas et al. from University Hospital Würzburg developed a new TNFR2 agonist, designated NewSTAR2, which was a single-chain encoded murine TNF80 trimer fused to the C-terminus of an irrelevant IgG1 molecule carrying the N297A mutation to avoid/minimize interaction with Fcγ-receptors (FcγRs) and improve serum retention. Single dose injection of NewSTAR2 resulted in a >3-fold increase of Tregs after 5 days of treatment. Tregs in NewSTAR2-treated mice exhibited enhanced suppressive activity, and upregulation of Treg markers such as CD39. Pretreatment with NewSTAR2 could protect mice from allogeneic hematopoietic cell transplantation-induced acute GvHD (Vargas et al.).

To date, the exact role of TNFR2 signaling in the outcome of transplantation of solid organ or islet cells remains elusive. Kouyoumdjian et al. from McGill University thoroughly analyzed the published data and concluded that TNFR2 signaling is beneficial in kidney and islet transplantation. Nevertheless, the roles of TNFR2 signaling and its potential therapeutic value in other types of solid organ transplantation should be further clarified by future studies (Kouyoumdjian et al.).

Skartsis et al. from University of California San Francisco explored the effects of pro-inflammatory factors on hTregs. They found that the treatment with IL-6 and TNF enhanced proliferation of Tregs in a TNFR2-dependent manner, without causing lineage destabilization. The results prompted the authors to develop a protocol using TNF, IL-6 and CD28 super-agonist to expand the functional Tregs *ex-vivo* for adoptive Treg transfer therapy (Skartsis et al.).

## Differential responses of Tregs and Teffs to TNFR2 co-stimulation at transcriptomic and metabolic levels

TNFR2 can be expressed by the activated T effector cells (7). Mensink et al. from Leiden University showed differential responses of human Tconvs (CD4^+^ conventional T cells) and tTreg (thymus derived Tregs) to TNFR2 co-stimulation at the transcriptomic and metabolic levels. Their results showed that both activated human Tconvs and tTregs could respond to TNFR2 stimulation, but in a different fashion, with the breadth of the transcriptomic changes much larger in tTregs. This suggests a greater impact of TNFR2 co-stimulation on the transcriptome of tTregs versus that of Tconvs. TNFR2 could also play a role in the metabolism of both tTregs and Tconvs. Its signal could remodel glutamine metabolism in both cell types. However, TNFR2 only enhanced glycolysis and TCA cycle in tTreg (Mensink et al.).

Taken together, the 9 publications in this Research Topic include translational research, mechanistic studies, meta-analysis and literature analysis. They showcase major directions and aim to help the research community to understand current progress in this rapidly growing field. Currently, a number of TNFR2-targeting antibodies are under clinical investigation, including those with both antagonistic or agonistic properties. The publications in this Research Topic also help set up the context to understand the clinical trial results which are expected to become available in the near future.

## Author contributions

All authors listed have made a substantial, direct and intellectual contribution to the work, and approved it for publication.
